# Che-1 sustains hypoxic response of colorectal cancer cells by affecting Hif-1α stabilization

**DOI:** 10.1186/s13046-017-0497-1

**Published:** 2017-02-18

**Authors:** Tiziana Bruno, Mariacristina Valerio, Luca Casadei, Francesca De Nicola, Frauke Goeman, Matteo Pallocca, Valeria Catena, Simona Iezzi, Cristina Sorino, Agata Desantis, Cesare Manetti, Giovanni Blandino, Aristide Floridi, Maurizio Fanciulli

**Affiliations:** 1 UOSD SAFU, Department of Research, Diagnosis and Innovative Technologies, Translational Research Area, Regina Elena National Cancer Institute, Via E. Chianesi 53, 00144 Rome, Italy; 2 Department of Chemistry, “Sapienza” University, Rome, Italy; 3 UOSD Oncogenomic and Epigenetic Unit, Regina Elena National Cancer Institute, Rome, Italy

**Keywords:** Che-1/AATF, Hypoxia, Metabolism, HIF-1α, PHD3/EGLN3, SIAH-2

## Abstract

**Background:**

Solid tumours are less oxygenated than normal tissues. Consequently, cancer cells acquire to be adapted to a hypoxic environment. The poor oxygenation of solid tumours is also a major indicator of an adverse cancer prognosis and leads to resistance to conventional anticancer treatments. We previously showed the involvement of Che-1/AATF (Che-1) in cancer cell survival under stress conditions. Herein we hypothesized that Che-1 plays a role in the response of cancer cells to hypoxia.

**Methods:**

The human colon adenocarcinoma HCT116 and HT29 cell lines undepleted or depleted for Che-1 expression by siRNA, were treated under normoxic and hypoxic conditions to perform studies regarding the role of this protein in metabolic adaptation and cell proliferation. Che-1 expression was detected using western blot assays; cell metabolism was assessed by NMR spectroscopy and functional assays. Additional molecular studies were performed by RNA seq, qRT-PCR and ChIP analyses.

**Results:**

Here we report that Che-1 expression is required for the adaptation of cells to hypoxia, playing an important role in metabolic modulation. Indeed, Che-1 depletion impacted on HIF-1α stabilization, thus downregulating the expression of several genes involved in the response to hypoxia and affecting glucose metabolism.

**Conclusions:**

We show that Che-1 a novel player in the regulation of HIF-1α in response to hypoxia. Notably, we found that Che-1 is required for SIAH-2 expression, a member of E3 ubiquitin ligase family that is involved in the degradation of the hydroxylase PHD3, the master regulator of HIF-1α stability.

**Electronic supplementary material:**

The online version of this article (doi:10.1186/s13046-017-0497-1) contains supplementary material, which is available to authorized users.

## Background

Hypoxia, i.e. low levels of O_2,_ is a common feature of solid tumours, which leads to an aggressive tumor phenotype. Hypoxia is also associated with resistance to therapeutic treatments and with a poor clinical prognosis [1]. The main adaptation of tumor cell to hypoxia is the switch from an aerobic metabolism to glycolysis [2].

The glycolytic pathway produces only 2 ATP (adenosine triphosphate) per molecule of glucose, but cancer cells compensate this reduced yield in ATP production increasing, by approximately ten fold the glycolytic flux of glucose to pyruvate, and then to lactate [3]. Glycolysis also provides precursors for the synthesis of nucleic acids, lipids and amino acids that are crucial to enhance survival and growth of cancer cells both during carcinogenesis, and in metastatic tumours [4, 5].

The master regulator of hypoxia response is the hypoxia inducible factor (HIF) family of transcription factors, in particular HIF-1α [6]. Indeed, in low oxygen tension HIF-1α is quickly stabilized and affects most of the cancer hallmarks involved in the hypoxic response [6]. This transcription factor regulates the expression of multiple genes involved in proliferation, apoptosis, immune response, genomic instability, pH homeostasis, invasion and metastasis [6] so that, as a rule, over-expression of HIF-1α is associated with a poor clinical outcome [7, 8]. HIF-1α expression is mainly regulated through oxygen-dependent proteosomal degradation. Under normoxic conditions, specific prolyl-hydroxylase domain proteins (PHD) hydroxylate HIF-1α, at one or both conserved prolyl residues, promote the binding of HIF-1α to the von Hippel Lindau protein (pVHL)-E3-ubiquitin ligase complex and then consequently its ubiquitination and degradation [9]. The inhibition of PHD enzymes by specific molecules leads to stabilization of HIF-1α, and constitutes a valid therapeutic approach in treating conditions of tissue stress, like inflammatory or ischemic events [10]. Importantly, under hypoxic conditions HIF-1α activity contributes to shift cell glucose metabolism from oxidative to glycolytic, by increasing the expression of glucose transporters (SLC2A1 and SLC2A3) and downstream glycolytic enzymes, making glycolysis the main source of energy in hypoxic cells [11, 12].

Several studies have been conducted to investigate how to reduce the ability of cells adapting to hypoxic conditions, where HIF-1α has emerged to be an attractive target for new anti -cancer therapies to improve the current treatments of metastatic and resistant cancers [13].

Che-1/AATF (Che-1) is a RNA polymerase II-binding protein mainly involved in different and fundamental processes in controlling transcription, cell cycle regulation, DNA damage response, and apoptosis [14]. This protein is regulated by several post-translational modifications, and in response to several kinds of stress, Che-1 not only activates p53 expression, but it also binds this onco-suppressor in such way that it represses the apoptotic arm of the p53 response [15, 16]. Moreover, Che-1 sustains mutant p53 expression, contributing to the growth of tumor cells [17]. More recently, Che-1 was found to be phosphorylated in response to several stresses, regulating mTORC1 and mTORC2 activity and activating autophagy, thus providing a new link between different cellular stress factors and mTOR signalling [18]. In this study, we explored the role of Che-1 in hypoxic response. We found that Che-1 expression is required for the adaptation of cancer cells to hypoxia. In particular, our results demonstrated that Che-1 depletion in the presence of hypoxia strongly reduces metabolic adaption and induces cell death. Taken together these findings reinforce the notion that Che-1 could be an attractive target for cancer therapy.

## Methods

### Cell line and culture conditions

HCT116 colon cancer cells were cultured in Dulbecco’s Modified Eagle Medium High Glucose (DMEM), HT29 and A549 cancer cell lines in RPMI 1640, supplemented with 10% fetal bovine serum (FBS). Cells were plated in 60 or 100 mm^2^ dishes and maintained at 37 °C in a humidified atmosphere of 5% CO_2_–95% air. For hypoxia treatments, cell culture dishes were exposed to a mix of 1% O_2_ saturated with 5% CO_2_ and 94% N_2_ for the time indicated, using hypoxia modulator chamber (Billups-Rothenberg). The temperature in the anaerobic chamber was set at 37 °C. The pH in the cell culture medium was determinated by CyberScan pH 510 Bench pH/mV Meter with ATC probe, integral electrode holder & power adapter (Thermofisher).

### Transfections, western blot analysis and antibodies

Che-1 siRNA was performed by transfecting a specific pool of double-stranded RNA oligonucleotides (siChe-1) (Stealth, Life Technologies-Thermofisher). Stealth siRNA Negative Control oligos were purchased from Life Technologies (Thermofisher). Transfections were carried out by Lipofectamine 3000 (Life Technologies-Thermofisher) in accordance with the manufacturer’s instructions.

Cell extract purifications and western blotting analyses were performed as previously described [18] by using the following antibodies: anti-Che-1 [19], anti-β-actin (Sigma, A5441, clone AC-15); anti-HIF-1α (BD Transduction Laboratories-610958); anti-HIF-1α (Bethyl Laboratories A300-286A); anti-Caspase-7 (Cell Signaling Technologies-12827); anti-EGLN3/PHD3 (Novus Biologicals NB100-139); anti-SIAH-2 (N14) (Santacruz sc-5507). Densitometric analyses of immunoblots were performed using Alliance system by UVITECH Cambridge, and Ratios were calculated by the following formula:

intensity sample/intensity β Actin

intensity control/intensity β Actin

### Assay of oxygen consumption

Cells were plated in 15 cm^2^ dishes and after 24 h they were transfected by specific siRNA. Then, the cells were exposed to hypoxia for 16 h. At the end of incubation, cells were trypsinized and suspended in DMEM at a concentration of 1 × 10^8^/ml. Oxygen consumption was measured with a Clark oxygen electrode (Yellow Spring Instruments Company) at 37 C°; the concentration of dissolved oxygen was taken to be 406 ng-atoms O/ml [20]. The rate of oxygen consumption was determined by adding 0.1 ml of cell suspension (1 × 10^7^ cells) to 1.9 ml of DMEM in a closed glass chamber of 2 ml capacity (Gilson Medical Electronics). The final concentrations of oligomycin, FCCP and antimycin A were respectively, 1 μg/ml, 0.25 μM, and 2 μM.

### Hexokinase activity assay

Cells were transfected with siControl or siChe-1, and after hypoxia treatment collected and processed. Cells were lysed and the activity of the Hexokinase was measured in accordance with the Hexokinase Assay Kit protocol (Cat# E-111) from Biomedical Research Service Center, using a SpectraMax M2 MultiMode Microplate Reader (Molecular Devices, Sunnyvale, CA, USA), with OD 492 nm. The activity was calculated in IU/L unit.

### Chromatin immunoprecipitation assay (ChIP)

Chromatin immunoprecipitation assays in HCT116 cells were performed as previously described [17], by using anti-HIF-1α (Bethyl). Immunoprecipitations with no specific immunoglobulins (Santa Cruz) were performed as negative controls. For quantitative ChIP analysis (ChIP-qPCR), 1ul of purified DNA was used for amplification on an Applied Biosystems 7500 Fast Real Time PCR system (with Applied Biosystem SYBR GREEN). The following human promoter-specific primers were employed in PCR amplifications:

PDK1 forward 5′- GAGCCTTTTGGCTGAGATTG -3′

PDK1 reverse 5′- GATGGGACTGGGGACACTAA -3′

PFKFB4 forward 5′- CCCTAGCAAGGAGGTAGCAG -3′

PFKFB4 reverse 5′- AGGCCAGGATCGAGAATGCG -3′

### RNA isolation and quantitative RT–PCR analysis

Total RNA was isolated using TRIzol reagent (Life Technologies-Thermofisher) in accordance with the manufacturer’s instructions, and the first-strand cDNA was synthesized with random primers and M-MLV reverse transcriptase (Life Technologies-Thermofisher). The cDNA was used for quantitative real-time PCR (qRT–PCR) experiments carried out in a 7500 Fast Real Time PCR System (Applied Biosystem-Thermofisher) using SYBR GREEN PCR Master Mix (Applied Biosystem-Thermofisher). ΔΔCt values were normalized with those obtained from the amplification of the endogenous RPL19 gene. Data are presented as the mean ± SD from three independent experiments performed in duplicate. The following human-specific primers were employed in PCR amplifications:

RPL19 forward 5′-CGGAAGGGCAGGCACAT -3′

RPL19 reverse 5′- GGCGCAAAATCCTCATTCTC -3′

Che-1 forward 5′- CCGGAATTCGGATAAGACCAAACTGGCT -3′

Che-1 reverse 5′- CCGCTCGAGGAGTTCTCGAAGGAGCTG -3′

SLC2A1 forward 5′- GTGGGAGGAGCAGTGCTTGG -3′

SLC2A1 reverse 5′- GACGATGCCCAGCTGGTGCA -3′

SLC2A3 forward 5′- TGCGGGGTGCCTTTGGCACT -3′

SLC2A3 reverse 5′- TGGGCTGTCGGTAGCTGGAC -3′

PFKFB4 forward 5′- GCGCATTGAGTGCTATGAGA -3′

PFKFB4 reverse 5′- AATATACGATGCGGCTCTGG -3′

PDK1 forward 5′- GCCCAGGGTGTGATTGAATA -3′

PDK1 reverse 5′- GGACTTCCTTTGCCTTTTCC -3′

SIAH-2 forward 5′- CATCAGGAACCTGGCTAT -3′

SIAH-2 reverse 5′- GGACGGTATTCACATATG -3′

PHD3 forward 5′- TCCCTGGGCTGGACTGACCTTT -3′

PHD3 reverse 5′- CCTCCCCCAAGAAGCCACTGAAA -3′

HIF1-α forward 5′- CCAGTTAGGTTCCTTCGATCAGT -3′

HIF1-α reverse 5′- TTTGAGGACTTGCGCTTTCA -3′

### ^1^H-NMR Spectroscopy

Each medium sample (2 ml) was lyophilized and then dissolved in 700 μl of 1 mM TSP [sodium salt of 3 (trimethylsilyl) propionic-2,2,3,3-d4 acid], 10 mM sodium azide D_2_O phosphate buffer solution (pH = 7.4) and finally homogenized by vortex mixing for 1 min. After centrifugation (10 min, 10.000 RCF at 22 °C), 600 μl of each resulting supernatant was transferred to a 5-mm NMR tube and used for the NMR analysis2D. 1H J-resolved (JRES) NMR spectra were acquired on a 500 MHz Varian/Agilent spectrometer (Agilent, Santa Clara, CA) using a double spin echo sequence with 4 transients per increment for a total of 32 increments. These were collected into 16 k data points using spectral widths of 6 kHz in F2 and 40 Hz in F1. There was a 2.0 s relaxation delay. Each FID was Fourier transformed after a multiplication with sine-bell/exponential function in the F2 dimension and a sine-bell function in the F1 dimension. JRES spectra were tilted by 45°, symmetrised about F1, referenced to TSP at δH = 0.0 ppm and the proton-decoupled skyline projections (p-JRES) exported using Agilent VNMRJ 3.2 software. Metabolites responsible for the separation between samples from cells treated with hypoxia in the presence or absence of siChe-1 were identified using an in-house NMR database and Chenomx NMR suite v. 7.7 (Chenomx Inc., Alberta, Canada).

### NMR spectra pre-processing treatment

The 1D skyline projections exported were aligned and then reduced into spectral bins with widths ranging from 0.01 to 0.02 ppm by using the ACD intelligent bucketing method (1D NMR Manager software (ACD/Labs, Toronto, Canada). To compare the spectra, the integrals derived from the binning procedure were normalized to the total integral region, following exclusion of bins representing the residual water peak (δ 4.33–5.17 ppm) and the TSP peak (δ 0.5–0.5 ppm). The resulting data was used as input for Principal Component Analysis (PCA) [21] and was performed by using SIMCA-P + version 12 (Umetrics, Umea, Sweden).

### RNA-Seq

HCT116 cell line was transfected with siChe-1 or siRNA negative control using Lipofectamine 3000 (Stealth Life Technologies-Thermofisher), and exposed to hypoxia for 4 h where indicated. Total RNA was extracted from cells using Trizol (Life Technologies-Thermofisher), purified and enriched by Qiagen RNeasy columns for gene expression profiling (Qiagen). Quantity and integrity of the extracted RNA were assessed by NanoDrop Spectrophotometer (NanoDrop Technologies) and by Agilent 2100 Bioanalyzer (Agilent Technologies), respectively. RNA libraries for sequencing were generated in accordance with the standard Illumina TruSeq RNA Library Prep Kit protocol using 2 μg total RNA as starting material. The resulting library was controlled qualitatively with the High Sensitivity DNA Kit (Agilent Technologies, Santa Clara, CA, USA) and quantitatively with real-time analysis employing a SYBR Green qPCR protocol with specific primers complementary to adapter sequences. Based on the qPCR quantification, libraries were normalized to 1 nM and denatured by using 0.1 N NaOH. Cluster amplification of denatured templates was carried out according to the manufacturer’s protocol (Illumina, Inc., San Diego, CA, USA). Sequencing was performed on a Genome Analyzer IIx (Illumina) in paired-end mode, sequencing from each side 51 bp. For each sample generated by the Illumina platform, a pre-process step for quality control was performed to assess sequence data quality and to discard low quality reads.

### Bioinformatic analysis

The analyses were performed by exploiting the RNA-seq analysis workflow RAP [22] that comprises of read mapping, transcript assembly and abundancy estimation followed by transcript-based differential expression via the Tuxedo suite [23]. Paired-end reads were mapped to the human genome assembly hg19 with TopHat and further analyzed by the Cufflinks-Cuffdiff pipeline to identify differentially expressed genes. We ran the pipeline without novel transcript discovery. Raw data (BAM files) were submitted to the National Center for Biotechnology Information (NCBI) Gene Expression Omnibus database (http://www.ncbi.nlm.gov/geo), with accession ID GSE90599. UCSC Genome Browser screenshots were created through loading bigWig files generated from the accepted_hits Tophat BAM file. Scatterplots were created thanks to the CummeRbund library. Custom plots such as barplots were created via custom R scripting.

### Statistical analysis

Statistical analyses were performed by using the Student two-tailed *t*-test to compare in vitro experiments. All statistical tests were carried out using GraphPad Prism version 5.0 for Windows, Graphpad Software, San Diego California USA (www.graphpad.com). Probability value of <0.05 was considered statistically significant.

## Results

### Che-1 protects colon cancer cells from apoptosis induced by hypoxia

**Fig. 1:**
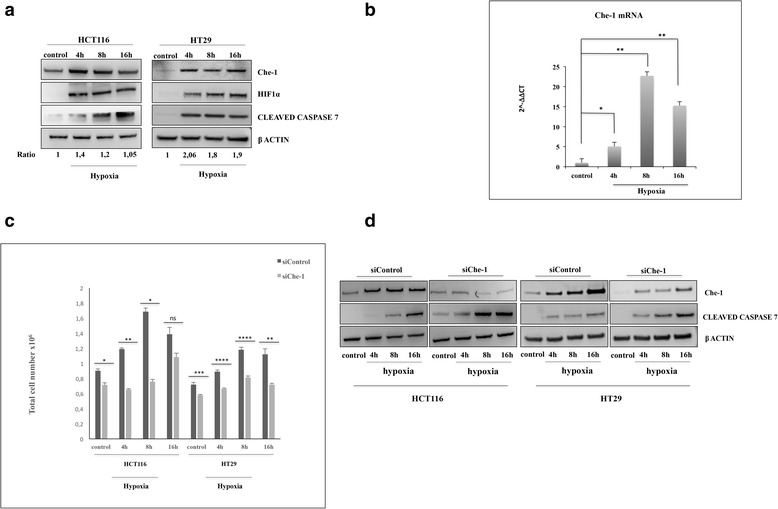
Che-1 is involved in the response to hypoxia. **a** Western blot (WB) analysis performed with the indicated antibodies (Abs) of total cell extracts (TCEs) from HCT116 or HT29 cells exposed to hypoxia (1% O_2_) for the times indicated_._ **b** Quantitative RT–PCR (qRT–PCR) performed in HCT116 cells exposed to hypoxic conditions as in **a**. Values were normalized to RPL19 expression. Bars represent the standard error of three different experiments. *P ≤ 0,02, **P ≤ 0.002. **c** HCT116 and HT29 cells were transiently transfected with Stealth siRNA negative control (siControl) or siRNA Che-1 (siChe1) and treated as in **a**. Cell proliferation was measured by counting the cells daily. A representative histogram is depicted utilizing values from three independent experiments and significance was calculated. *P ≤ 0.02, **P = 0.0005, ***P = 0.0008, ****P ≤ 0,0001, n.s., not significant. **d** WB analysis with the indicated Abs of TCEs from HCT116 and HT29 cells transiently transfected and treated as in **c**

Over the last few years, Che-1 has been identified as a protein involved in several cellular pathways including the control of transcription and apoptosis [14]. In particular, several findings indicate that Che-1 is involved not only in DNA damage response (DDR) but also in other types of stress. Indeed, it has been more recently documented that Che-1 protects cells from apoptosis induced by ionizing radiation, glucose deprivation and hypoxia, inducing autophagy by the control of the kinase activity of mTOR [18]. In order to better investigate the role of Che-1 in response to hypoxia, we exposed HCT116 and HT29 colon cancer cells to low tension of oxygen at different times. The hypoxic treatment at 4 h induced, together with HIF-1α stabilization, an increase of Che-1 levels (Fig. 1a) through its phosphorylation [18], whereas apoptosis activation after 8 h, but mostly after 16 h, induced a degradation of Che-1 as already observed with other forms of stress (Fig. 1a). In addition, we analyzed Che-1 mRNA levels, demonstrating that hypoxia is able to regulate the expression of this gene also at transcriptional level (Fig. 1b). In order to better understand the involvement of Che-1 in hypoxia response, we depleted Che-1 expression by a specific pool of siRNA oligos. As shown in Fig. 1c and d, Che-1 depletion significantly decreased cell viability, strongly increasing apoptosis induction. Taken together these data demonstrate that Che-1 levels are regulated by hypoxia, and its expression is required for the cellular response to this kind of stress.

### Che-1 is required for metabolic adaption to hypoxia

**Fig. 2:**
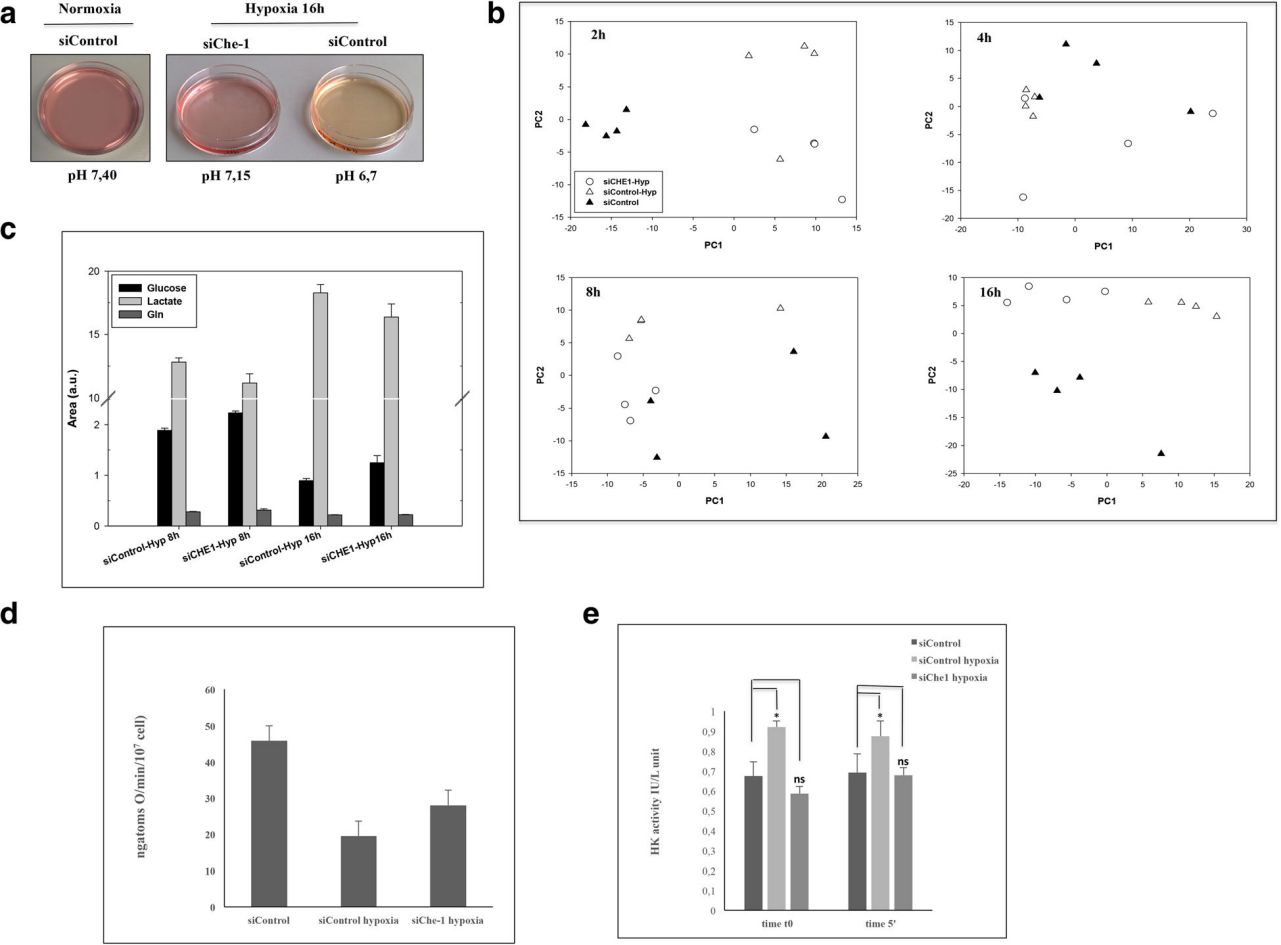
Che-1 is involved in the metabolic switch in response to hypoxia. **a** HCT116 cells were transiently transfected with stealth siRNA negative control (siControl) or siRNA Che-1 (siChe-1) and exposed to hypoxia for 16 h where indicated and pH was measured. **b** Score plots indicating metabolic differences between hypoxic and normoxic samples from HCT116 cells transiently transfected as in **a** and exposed to hypoxia for the times indicated. **c** Glucose, lactate and glutamine levels in HCT116 cells transiently transfected as in **a** and exposed to hypoxia for the indicated times. The levels are the means ± S.D. of four independent experiments *p* < 0.05). **d**-**e** HCT116 cells were treated as in A and Oxygen consumption (**d**) and HK activity (**e**) were measured. Values are mean of three independent experiments. *p < 0.05 (**d**) *p < 0.05, n.s., not significant (**e**)

It has been extensively demonstrated that the principal mechanism by which the cell protects itself after hypoxia is a switch of the glucose metabolism from mitochondrial respiration to glycolysis [2]. Based on the abovementioned considerations, we evaluated whether Che-1 depletion could affect this switch. The culture medium of cells lacking Che-1 expression, placed in a hypoxic environment rapidly becomes more acidic due to high production of lactic acid, compared to control cells. As shown in Fig. 2a, after 16 h of hypoxia, HCT116 siControl cells showed a much lower pH compared to the cells under normoxic conditions (6,7 vs 7,40). On the contrary, the pH level of Che-1 depleted cells after 16 h of hypoxia was similar to normoxic cells (7,15 vs 7,40), thus suggesting an involvement of Che-1 in this metabolic switch. Similar results were obtained when we analyzed two other cell lines (Additional file 1: Figures S1A and B). To further investigate these findings, we analyzed by using PCA ^1^H NMR spectra collected on medium samples from HCT116 cells treated or untreated with hypoxia at different times, in the presence or absence of siChe-1 (Fig. 2b). At an earlier time point, we only discerned a significant metabolic difference between hypoxic and normoxic samples (*p* < 0.0001 on PC1). Notably, after 4 hours of hypoxic treatment, we observed that the metabolism of Che-1 depleted cells was different from that of their hypoxic and normoxic controls (*p* = 0.035 on PC2). Moreover, at this time, we did not observe any significant differences between the two groups of controls. As the experimental times elapsed, the metabolic effects due to the depletion of Che-1 became more evident: we observed metabolic differences between samples from Che-1 depleted cells and their control cells in hypoxic conditions at 8 h on PC2 (*p* = 0.003) and at 16 h on PC1 and PC2 (*p* = 0.002). It is important to note that the metabolism of Che-1 depleted cells were very similar to that of their controls under normoxic conditions. Indeed, the difference between Che-1 depleted cells in hypoxic condition and control cells in normoxic condition did not result significant at 8 h, resulting significant only at 16 h on PC2 (*p* = 0.002). Conversely, the metabolic effects of hypoxia were more evident on cells without depletion of Che-1: the differences between controls in hypoxic or normoxic conditions were evident at 8 (*p* = 0.010 on PC2) and 16 h (*p* = 0.016 on PC1 and *p* = 0.003 on PC2). These results were further confirmed by analyzing HT29 and A549 cells (Additional file 1: Figures S1C and D). Subsequently, in order to identify the main metabolites contributing to the discrimination between Che-1 depleted cells and their control in hypoxic conditions at 16 h on PC1, we analyzed the loadings values from PCA with a threshold of 0.95. PC1 included the following variables with the highest correlation levels: glucose with positive loadings and lactate with negative loadings. Therefore, considering the net balance of these metabolites, PC1 indicates that the depletion of Che-1 induced a lower consumption of glucose as well as a lower production of lactate (Fig. 2c). The reduced production of lactate in Che-1 depleted cells were further confirmed in HT29 cells (Additional file 1: Figure S1E). Moreover, the absence of a significant variation in the consumption of glutamine between HCT116 cells under hypoxic conditions with or without depletion of Che-1 suggested that the significant difference in lactate production was only correlated to glucose consumption, and therefore that Che-1 depletion decreases the glycolytic phenotype of HCT116 cells under hypoxic conditions. These findings were confirmed by an increase of oxygen consumption (Fig. 2d), and a decrease in Hexokinase (HK) activity (Fig. 2e), in cells depleted for Che-1. Taken together, these results demonstrate an important role of Che-1 in the metabolic adaptation in response to hypoxic stress.

### Che-1 modulates genes involved in the response to hypoxia

**Fig. 3:**
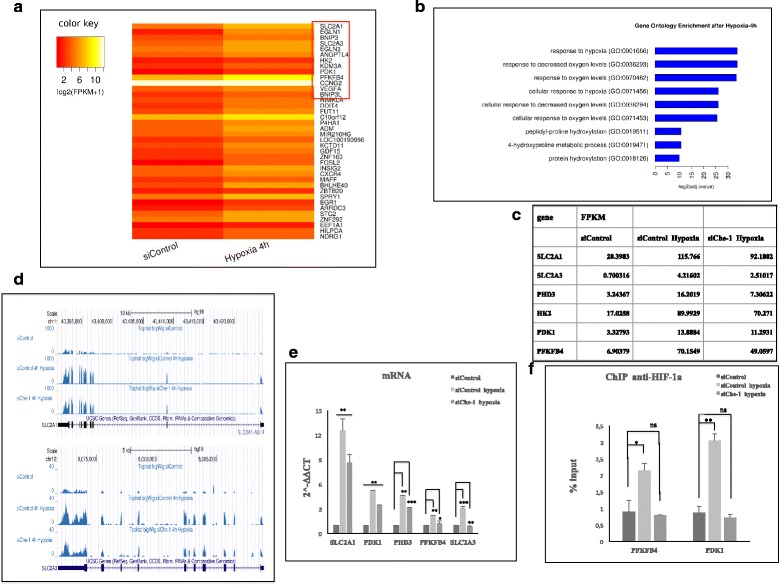
Che-1 regulates genes transcription in response to hypoxia. **a** Intensity Heatmap of modulated genes in HCT116 cells after 4 h of exposure to hypoxia highlighted by *red* frame. **b** Gene Ontology from Enrichr [42]. Calculated from the most significant (corrected P <0.05) 38 genes regulated in Cuff diff analysis, Normoxic cells VS Hypoxic cells. **c** Expression levels in HCT116 cells transiently transfected with Stealth siRNA negative control (siControl) or siRNA Che-1 (siChe-1) and exposed to hypoxia for 4 h. Gene expression levels are reported as FPKM reconstructed from Cufflinks RNA-Seq analysis. **d** Genome Browser screenshot of Big Wig Enrichment from RNA-Seq BAM files, showing expression of SLC2A1 (right) and SLC2A3 (left) genes in HCT116 cells transiently transfected with Stealth siRNA negative control (siControl) or siRNA Che-1 (siChe-1) and exposed to hypoxia for 4 h. **e** Quantitative RT–PCR (qRT–PCR) for metabolic genes expression was performed in HCT116 cells transiently transfected and treated as in **c**. Values were normalized to RPL19 mRNA expression. Error bars represent the standard error of three different experiments. *P ≤ 0,006, **P ≤ 0.008, ***P ≤ 0.0008. **f** ChIP-qPCR analysis of HCT116 cells transfected with siControl or siChe-1 exposed to hypoxia, using anti-HIF-1α Ab or control IgGs. Error bars represent the standard error of three different experiments. *P ≤ 0.004, **P = 0.0005

Since Che-1 is an important transcriptional co-factor, we wondered whether this protein regulated glucose metabolism by affecting the transcriptional activation in response to hypoxia. To address this hypothesis, we performed a whole transcriptome sequencing (RNA-seq) analysis in HCT116 cells treated or untreated with hypoxia, in the presence or absence of Che-1 expression. Differential Gene Expression analysis with standard parameters (corrected *p*-value < 0.05) showed 38 modulated genes after 4 h of hypoxia treatment (Fig. 3a), and as expected, Gene Ontology analysis of these genes revealed a strong enrichment of several gene clusters related to cellular metabolic processes (Fig. 3b). Among them, we observed that Che-1 depletion was able to modulate genes playing a key role in glucose transport (SLC2A1 and SLC2A3) and glucose metabolism (PFKFB4, PDK1, PHD3) (Fig. 3d-e-f and Additional file 2: S2A). Notably these genes are finely regulated by HIF-1α in response to hypoxia [11–25]. This observation prompted us to evaluate whether Che-1 could affect HIF-1α recruitment onto the promoters of these target genes. To do this, we performed ChIP experiments, immunoprecipitating HIF-1α in cells depleted or not for Che-1 and exposed to hypoxia. In absence of Che-1 expression, the analysis of PDK1 and PFKFB4 promoters, revealed a strong decrease of HIF-1α recruitment, thus confirming an involvement of Che-1 in HIF-1α activity (Fig. 3f). Taken together, these results provide strong evidence that Che-1 is required by HIF-1α to regulate target genes involved in metabolism.

### Che-1 promotes the degradation of HIF-1α through the transcriptional regulation of the RING E3 ubiquitin ligase SIAH2

**Fig. 4:**
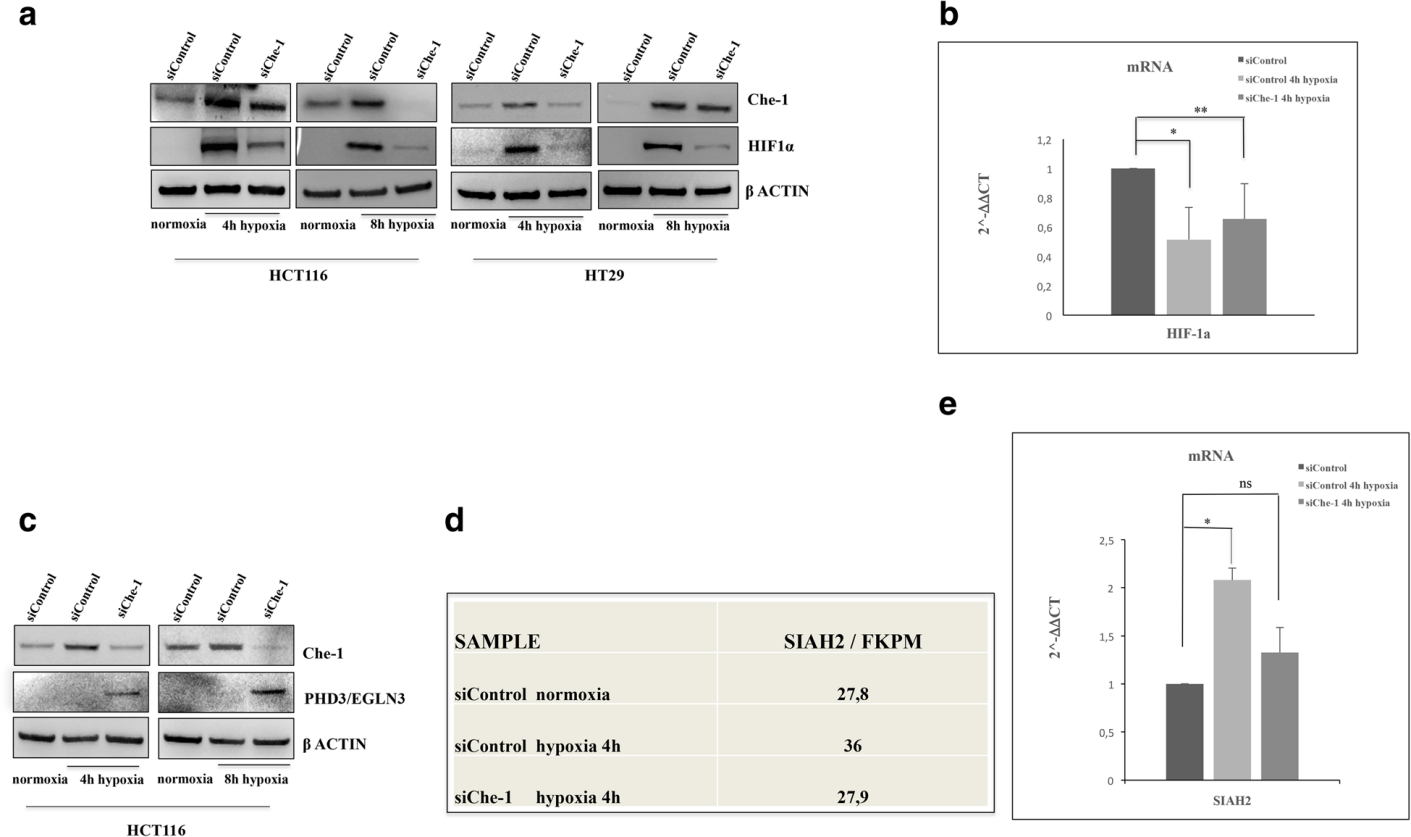
Che-1 modulates HIF-1α protein expression. **a** WB analysis with the indicated Abs of TCEs from HCT116 and HT29 cells transiently transfected with stealth siRNA negative control (siControl) or siRNA Che-1 (siChe-1) and exposed to hypoxia where indicated. **b** qRT–PCR performed in HCT116 cells transiently transfected and treated as in **a**. Values were normalized to RPL19 mRNA expression. Error bars represent the standard error of three different experiments. *P ≤ 0,02, **P ≤ 0.01. **c** WB analysis with the indicated Abs of TCEs from HCT116 cells transiently transfected as in **a**. **d** SIAH-2 expression levels are reported as FPKM reconstructed from Cufflinks RNA-Seq analysis from HCT116 transiently transfected with siControl or siChe-1 and exposed to hypoxia. **e** qRT–PCR performed as in **b**. Values were normalized to RPL19 mRNA expression. Error bars represent the standard error of three different experiments. *P ≤ 0,02, n.s., not significant

Next, we attempted to shed light on the mechanism/s by which Che-1 exerts its activity on HIF-1α functions. For this purpose, we initially tested the possibility of a physical interaction between these proteins, but immunoprecipitation experiments did not show any binding between them (data not shown). Therefore, we examined whether Che-1 was able to regulate HIF-1α expression in HCT116 and HT29 cells undepleted or depleted for Che-1, and exposed them to hypoxia at different times. As shown in Fig. 4a, HIF-1α expression was undetectable under normoxic conditions, but its protein levels markedly increased during hypoxic treatment, where instead in Che-1 depleted cells this phenomenon was strongly reduced. The analysis of HIF-1α mRNA expression under the same conditions revealed that, unlike the reduction observed at the protein level, there was no significant decrease of HIF-1α transcription in Che-1 depleted cells (Fig. 4b), suggesting the presence of another mechanism for its regulation. HIF-1α is a central regulator of the cellular response to hypoxia, and its expression is strongly regulated through prolyl-hydroxylation by PHD enzymes required for its degradation. In particular, PHD3 exhibits high affinity to hydroxylase HIF1α [26]. This enzyme is also a target of HIF-1α and its expression is regulated in response to hypoxia [27–29]. Notably, Che-1 depletion induced a significant increase of PHD3 in cells exposed to hypoxia, in agreement with the observed decrease of HIF-1α levels (Fig. 4a–c). Thus, we analyzed the regulation of PHD3 expression in response to hypoxic treatments. Since RNA–Seq analysis demonstrated that PHD3 mRNA expression is in fact increased by hypoxic conditions and Che-1 depletion induces a reduction of its/this activation (Fig. 3d–f), we evaluated if Che-1 could exert its activity at the post-transcriptional level. In attempting to characterize this phenomenon, we focused our attention on the RING finger E3 ligase SIAH, a family proteins able to induce the ubiquitination and degradation of a several substrates, regulating in this way, different pathway and numerous biological processes. SIAH2 like other ubiquitin ligases can promote its own degradation and it is generally present at very low levels in cells generating many problems to detect the levels of this protein [30–32]. Previous work demonstrated that SIAH2 is also able to modulate PHD3 level in response to hypoxia, affects its stability [30]. As shown in Fig. 4d, in hypoxic condition, Che-1 depletion produced a decrease of SIAH-2 mRNA expression, indicating that the transcription of this gene is modulated by Che-1 activity. These results were confirmed by a qRT-PCR analysis, showing a decrease of the levels of SIAH-2 transcript concomitantly to Che-1 inhibition (Fig. 4e). Collectively, these findings demonstrate that Che-1 plays an important role in HIF-1α stabilization, consequently strongly affecting metabolism regulation in response to hypoxia.

## Discussion

In response to hypoxia, the cell has devised numerous adaptation strategies enabling it to survive. These mechanisms are mainly implemented at transcriptional level, through the activation and stabilization of the transcription factor HIF-1α [6]. Its activity is mainly reflected in a change of glucose metabolism, which switches from mitochondrial respiration to increased anaerobic glycolysis [33].

In this work we show how Che-1 plays an important role in the adaptation of the cell to hypoxia. Indeed, Che-1 results activated after hypoxic treatment in HCT116 and HT29 cells (Fig. 1a), and its depletion increases apoptosis following hypoxia, underlying the relevance of Che-1 in cell survival (Fig. 1c and d). Our group, as well as other researchers, has previously shown how numerous types of stress affect the half-life of Che-1, its localization and its functions [16–18]. These effects are mediated through specific Che-1 phosphorylations by DDR checkpoint kinases such as ATM, Chk2 and MK2 [24]. Although hypoxia does not induce DDR, it has been recently shown that it can still induce ATM activity [34], leaving to assume that even in these stressful conditions for the cell, this kinase may be responsible for the phosphorylation and stabilization of Che-1. Notably, unlike other cellular stresses such as genotoxic insult [35], in response to hypoxic stress Che-1 is also activated at the transcriptional level, especially during the early hours (Fig. 1b). These findings suggest that Che-1 may be a direct target of HIF-1α, but further studies will be needed to confirm this hypothesis and to characterize the mechanisms of this regulation.

Previous findings have demonstrated that hypoxia elicits alterations in cell metabolism through several mechanisms [36]. In this study we demonstrate an involvement of Che-1 in cell metabolic adaptation upon hypoxic conditions. Indeed, its depletion leads to an inhibition of the metabolic switch observed in cells subjected to hypoxic treatment (Fig. 2a and b). The relevance of the involvement of Che-1 in cell adaption is confirmed by the evidence that its depletion produces a markedly reduction of glucose and glutamine consumption (Fig. 2c), associated with reduced inhibition of oxygen consumption (Fig. 2d) and with less activation of glycolytic enzymes (Fig. 2e).

Hypoxia is one of the most important pathological characteristics of solid tumors, and is clinically associated with a poor prognosis as it may adversely affect the outcome of radiotherapy and chemotherapy [37, 38]. Another important feature of cancer cells is the high glycolytic activity even in the presence of normal oxygen level (Warburg effect) [39]. Our findings show how Che-1, in presence of hypoxia, is required for the production of primary metabolic substrates utilized from cancer cells for ATP production and macromolecules biosynthesis, needed for their proliferation and progression [40]. Preliminary results have shown that Che-1 depletion produces metabolic alterations in cancer cells even in absence of the hypoxic stress, leading us to speculate its possible role played in the Warburg effect [39, 41]. However, further experiments will be required to understand the Che-1’s true role in these complex pathways. Moreover, it also possible that the high levels of Che-1 observed in numerous tumor cell lines, not only contribute to cell proliferation, but also play an important role in the metabolic adaptation of these cells.

Che-1 exerts its functions mainly as a coactivator of transcription [14], and even in the case of cellular response to hypoxic stress, its depletion produces a decrease in the transcriptional activity of HIF-1α (Fig. 3). This might imply a direct action of Che-1 on transcription along with this transcriptional factor. However, although some preliminary experiments showed the presence of Che-1 on the promoters of genes regulated by HIF-1α (data not shown), co-immunoprecipitation experiments have failed to demonstrate the direct interaction between these two proteins.

Our results, however, show that Che-1 is required for HIF-1α stabilization in response to hypoxia (Fig. 4a). In fact, in Che-1 depleted cells there is a strong decrease in the protein levels of this transcription factor but not of its RNA messenger (Fig. 4b).

**Fig. 5:**
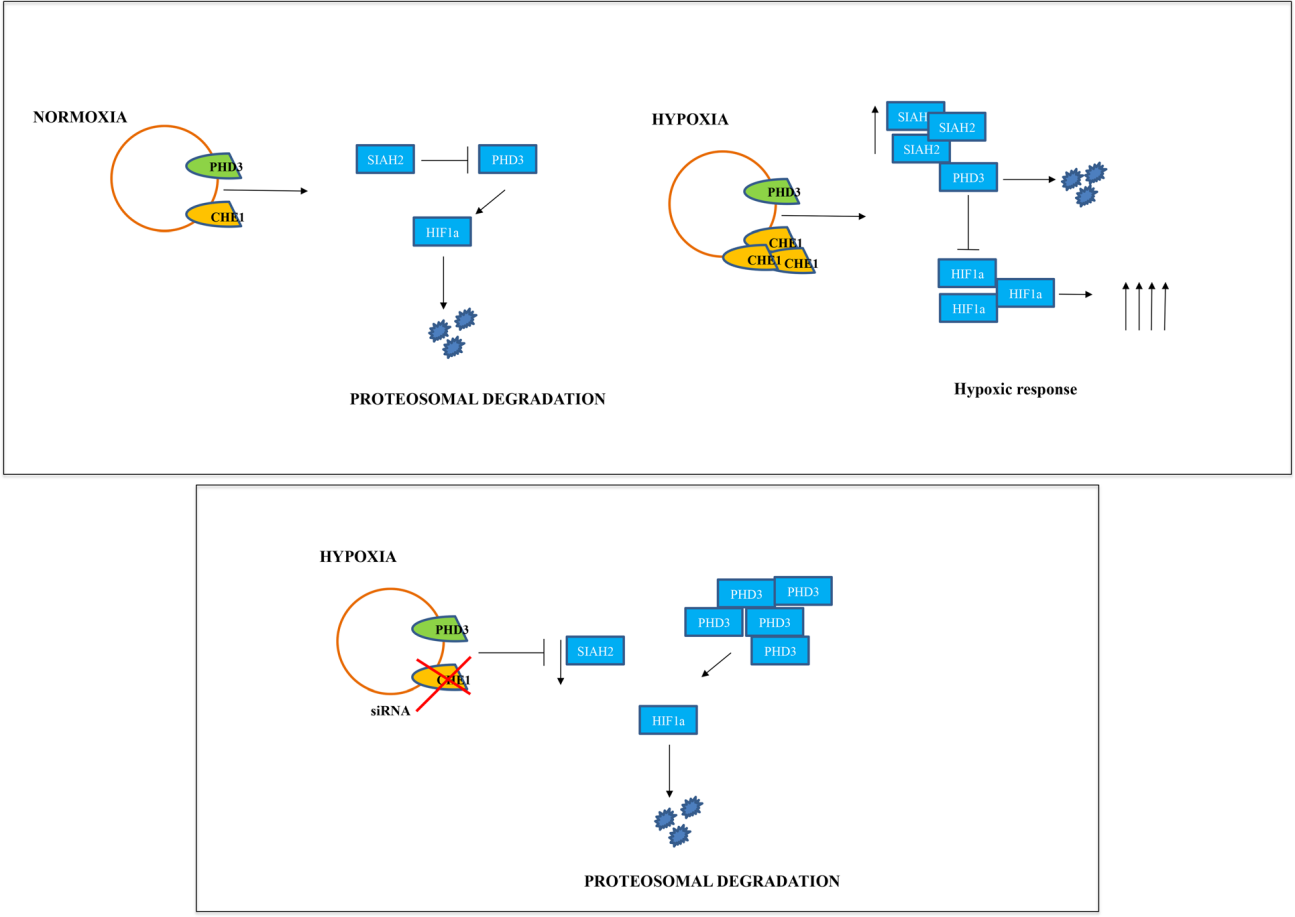
Model to explain Che-1 involvement in HIF-1α stabilization. Under hypoxic conditions the depletion of Che-1 leads to a decrease of the ubiquitin E3 ligase SIAH2, allowing an increase of the prolyl isomerase PHD3. This results in a decrease of HIF-1α stabilization (*bottom*)

All this appears to be the result of a minor transcription of SIAH-2 (Fig. 4c and d), necessary for the degradation of PHD3 [30]. This transcriptional downregulation of SIAH2 by Che-1 increase PHD-3 availability during hypoxia, providing a negative feedback mechanism for HIF-1α stability (Fig. 5). Evidently, we cannot rule out that Che-1 may either adjust the levels of HIF-1α also through other mechanisms, or affect the metabolism triggering other pathways. Indeed, only future studies will be able to shed light on these aspects.

## Conclusion

In conclusion, our data from these studies reveal a novel role for Che-1 as molecular determinant in the response to hypoxia of colorectal cancer cells. The ability of Che-1 to regulate HIF1-α stabilization, provides a novel metabolic target for tumor treatment.

## Additional files

Additional file 1: Figure S1. Che-1 is involved in the metabolic switch in response to hypoxia. A- and B- HT29 (A) and A549 (B) cells were transiently transfected with stealth siRNA negative control (siControl) or siRNA Che-1 (siChe-1) and exposed to hypoxia for 16 h where indicated and pH was measured. C- and D- Score plots indicating metabolic differences between hypoxic and normoxic samples from HT29 (C) and A549 (D) cells transiently transfected as in A. E- HT29 cells were transfected as in A and the medium lactate content was evaluated. (TIF 5841 kb)
Additional file 2: Figure S2. Che-1 regulates genes transcription in response to hypoxia. A- Quantitative RT–PCR (qRT–PCR) for metabolic genes expression was performed in HT29 cells transiently transfected with Stealth siRNA negative control (siControl) or siRNA Che-1 (siChe-1) and exposed to hypoxia for 4 h. Values were normalized to RPL19 mRNA expression. Error bars represent the standard error of three different experiments. **P* = 0,0010, ***P* ≤ 0,0003, ****P* ≤ 0,004, n.s., not significant. (TIF 1835 kb)

## Abbreviation

ATP: Adenosine triphosphate
ChIP: Chromatin Immuno-Precipitation
DDR: DNA Damage Response
HIF-1α: Hypoxia Inducible Factor 1α
HK: Hexokinase
NGS: Next Generation Sequencing
NMR: Nuclear Magnetic Resonance
O2: Oxygen
PC: Principal Component
PCA: Principal Component Analysis
PHD: Prolyl Hydroxylase Domain
pVHL: Protein Von Hippel Lindau
RAP: RNA-Seq Analysis Pipeline
RNA-seq: Ribonucleic acid sequencing
SIAH2: Seven in Absentia Homolog 2
SLC2A: Solute carrier family
