# Supplementary figures and images for: Che-1 sustains hypoxic response of colorectal cancer cells by affecting Hif-1α stabilization
Source: J Exp Clin Cancer Res. 2017 Feb 18;36:32. doi: 10.1186/s13046-017-0497-1 (PMC5316229; doi:10.1186/s13046-017-0497-1)

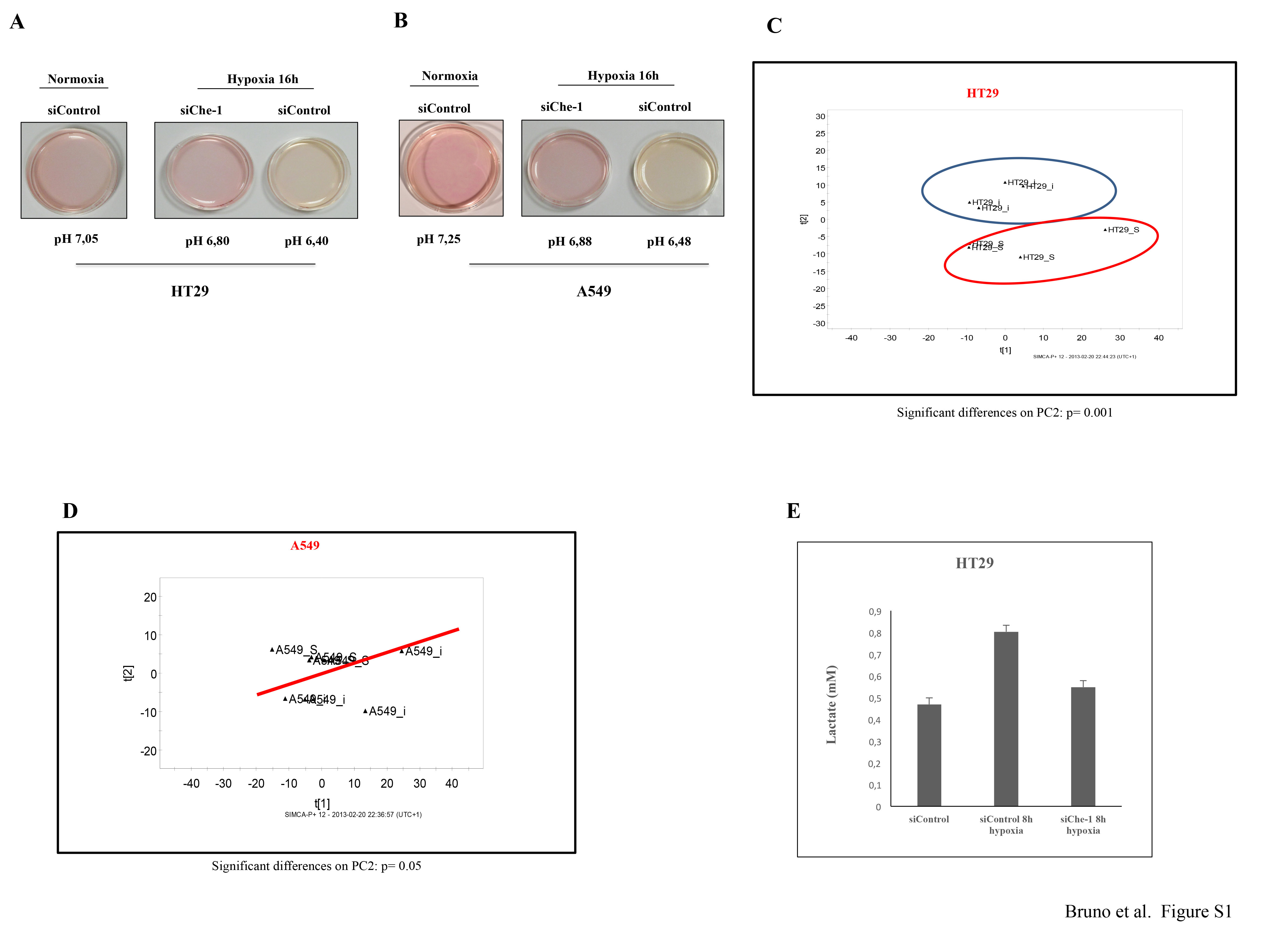

Supplement: Additional file 1: Figure S1. — Che-1 is involved in the metabolic switch in response to hypoxia. A- and B- HT29 (A) and A549 (B) cells were transiently transfected with stealth siRNA negative control (siControl) or siRNA Che-1 (siChe-1) and exposed to hypoxia for 16 h where indicated and pH was measured. C- and D- Score plots indicating metabolic differences between hypoxic and normoxic samples from HT29 (C) and A549 (D) cells transiently transfected as in A. E- HT29 cells were transfected as in A and the medium lactate content was evaluated. (TIF 5841 kb) [file 13046_2017_497_MOESM1_ESM.tif]

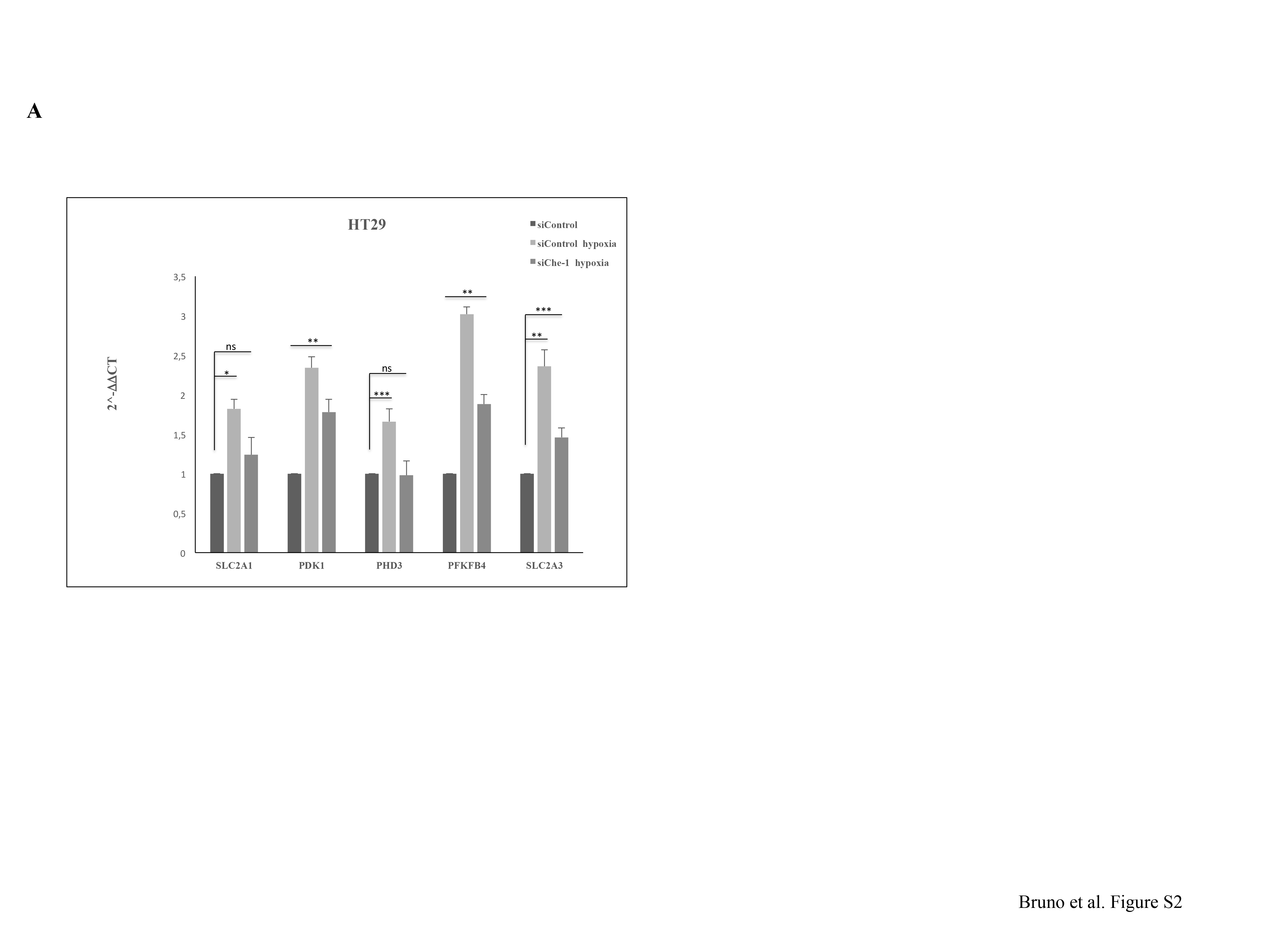

Supplement: Additional file 2: Figure S2. — Che-1 regulates genes transcription in response to hypoxia. A- Quantitative RT–PCR (qRT–PCR) for metabolic genes expression was performed in HT29 cells transiently transfected with Stealth siRNA negative control (siControl) or siRNA Che-1 (siChe-1) and exposed to hypoxia for 4 h. Values were normalized to RPL19 mRNA expression. Error bars represent the standard error of three different experiments. *P = 0,0010, **P ≤ 0,0003, ***P ≤ 0,004, n.s., not significant. (TIF 1835 kb) [file 13046_2017_497_MOESM2_ESM.tif]
